# Risk behaviors and well-being among Egyptian and Roma adolescents in Albania during the COVID-19 pandemic: Vulnerability and resilience in a positive youth development perspective

**DOI:** 10.3389/fpsyg.2023.989661

**Published:** 2023-08-01

**Authors:** Diana Miconi, Sonia Ouimet, Mohammed Abdullah Heel Kafi, Eglantina Dervishi, Nora Wiium, Cécile Rousseau

**Affiliations:** ^1^Department of Educational Psychology and Andragogy, Université de Montréal, Montréal, QC, Canada; ^2^Department of Psychology and Pedagogy, Faculty of Social Sciences, University of Tirana, Tirana, Albania; ^3^Faculty of Psychology, University of Bergen, Bergen, Norway; ^4^Division of Social and Cultural Psychiatry, McGill University, Montréal, QC, Canada

**Keywords:** Egyptian and Roma youth, positive development, COVID-19, risk behaviors, well-being, developmental assets, school drop-out, Albania

## Abstract

**Introduction:**

Egyptian and Roma communities represent two of the most deprived and stigmatized ethnic minorities in Albania. However, research investigating vulnerability and well-being in youth from these communities is scant. Even less is known among Egyptian and Roma adolescents who dropped-out of school. Within a Positive Youth Development framework, we investigated among Egyptian and Roma adolescents: (1) risk behaviors, well-being, and developmental assets (personal and contextual); (2) associations of developmental assets with risk behaviors and well-being; (3) specificities by ethnicity, gender, and education.

**Methods:**

A total of 201 Egyptian and Roma adolescents (*M_age_* = 16.63, *SD_age_* = 1.80; 47% girls; 53% school dropouts) completed a series of questionnaires in a community setting in August 2020 (first wave of the COVID-19 pandemic).

**Results:**

Binomial, Poisson and linear regression models indicated that Egyptian and Roma adolescents reported similar and high levels of risk behaviors, with boys reporting overall more risk behaviors than girls. Low level of well-being and of personal and contextual assets were reported. Girls reported higher family assets, positive values and social competencies than boys. The situation of adolescents attending school was overall not better than that of youth who had dropped out. Higher positive identity was associated with higher well-being.

**Discussion:**

Intervention and prevention efforts are urgently needed to support minority adolescents’ development during and in the aftermath of the pandemic. They should address the structural factors which limit the availability of personal and contextual resources in minority youth’s lives. Interventions aimed at building safer neighborhoods and providing safe access to schools for minority youth should be a priority and are essential to prevent the widening of inequalities during and after this health emergency.

## 1. Introduction

Egyptian and Roma communities are two of the most stigmatized and discriminated minority groups in Albania (De Soto et al., 2005). Due to systemic discrimination and inequalities, Egyptian and Roma youth’s voices and participation are often excluded from research, policies and interventions (Larkins, 2020). Indeed, Egyptian and Roma communities are extremely hard to reach compared to other minorities in Albania and this is partly due to their distrust of the majority group as well as some resistance to revealing their ethnicity because of fear of discrimination (Klaus and Marsh, 2014). Hence, the inclusion of Egyptian and Roma youth in research and intervention remains a challenge across European countries (Ringold et al., 2005), even more so during the present COVID-19 pandemic, which has highlighted and worsened social inequalities, impacting on a larger scale the health and psycho-social well-being of minority and racialized groups (Novacek et al., 2020; Miconi et al., 2021b). A recent report denounced a concerning situation during this health emergency for Roma communities across nine European countries. The authors found that these communities lacked essential and basic health, income and education, and that due to systemic discrimination, Roma youth were excluded from participation in research initiatives (Larkins, 2020). At the beginning of the pandemic, the United Nations Committee on the Rights of the Child’s highlighted the importance of the participation and inclusion of Roma’s youth in the research and response to the pandemic to prevent the widening of health, social and educational inequalities (Larkins, 2020; Milkova and Larkins, 2020). However, to date, little has been done in this direction.

In light of prior research showing that participation rates of Roma youth in research studies can be as high as 98% when trustful collaborations and alliances with local communities are established (Dimitrova et al., 2017b), the present study adopted a community-based approach along with a Positive Youth Development (PYD) perspective. PYD suggests that adolescents are not problems to be solved but resources to be developed and that they can thrive and subsequently actively contribute to and shape their personal development and that of their contexts of life as well as, ultimately, that of the broader society. Noteworthy, a PYD approach allows to adopt a resilience-oriented perspective to the study of minority youth that focuses not just on negative but also on positive outcomes, allowing to overcome the limitations of a more deficit-oriented approach that could in contrast contribute to the maintenance of negative stereotypes around adolescence and minority groups. Within the PYD literature, the developmental assets framework (Benson, 2007) is a theoretical model that has been empirically tested across multiple cultural contexts (Scales et al., 2017), representing a promising framework for cross-cultural research as well as for research across multiple ethno-cultural and minority groups. This framework suggests that the possibility for adolescents to rely on the alignment of resources in their contexts of life with their personal strengths can boost their well-being and reduce risk behaviors (Scales et al., 2017; Vazsonyi et al., 2020). Hence, developmental assets can represent a potential protective factor to be targeted by prevention and intervention programs.

Research on developmental assets and their association with well-being and risk behaviors among Egyptian and Roma youth is scarce. However, such research is crucial to empirically inform prevention and intervention efforts targeting these minority youth. In the current study, we investigate risk behaviors and well-being of Egyptian and Roma youth during the first wave of the COVID-19 pandemic in Albania as well as potential personal and contextual developmental assets able to promote youth’s adjustment. In addition, we explore whether youth’s risk behaviors, well-being and assets vary across ethnic groups, genders and between youth who are attending or completed secondary school and those who dropped-out of school. The focus on the strengths and resources of minority youth in a PYD perspective as well as the community-based approach align with social justice principles that aim at promoting empowerment and resilience in at risk communities while reducing the negative stigma that often originates from dominant research paradigms which adopt a more deficit-oriented perspective (Cabrera and Leyendecker, 2017; Leman et al., 2017; Motti-Stefanidi and García Coll, 2018).

### 1.1. Egyptian and Roma youth in Albania

Located in South-Eastern Europe, Albania is still one of the poorest countries in the Balkan region. Despite being now considered an upper-middle income economy (World Bank Group, 2021), the economic situation in the country is still very unstable, as shown by the very high youth unemployment rate (31%; Uka et al., 2021). The pandemic, as well as the devastating earthquake in 2019, have further compromised the socio-economic development of the country (World Bank Group, 2021). At the time of the study (August 2020), youth were living in a partial de-confinement situation after a very strict lockdown put in place to slow down rates of COVID-19 infections in the country (Open Data, 2021).

In the present study we refer to Roma communities and acknowledge the diversity and multiplicity of identities within this group (Parekh and Rose, 2011). The Roma ethnic group is one of the largest and most vulnerable minority groups in Europe, with an estimated population of 10 to 12 million (European Union, 2008). The Roma communities left India in the 11th century to settle primarily in Central and Eastern Europe (Vermeersch and Ram, 2009) and have a long history of persecution and marginalization.

Egyptian communities are considered distinct from the Roma and Albanian communities, due to their roots as descendants of Egypt and their own cultural traditions (Home Office, 2017). Nonetheless, research on Egyptian youth is particularly scarce, as they are often merged with either Albanian or Roma groups in surveys, when not completely excluded from research efforts. The few available data on these communities in Albania suggest that their situation is concerning and very similar to that of Roma communities, as they face some of the same barriers in society (De Soto et al., 2005; Republic of Albania, 2015).

Indeed, beyond their historical and cultural specificities, Egyptian and Roma minorities are two of the most stigmatized ethnic minorities in Albania and share a very concerning situation. They face many challenges, such as poverty, discrimination and stigmatization (De Soto et al., 2005; Republic of Albania, 2015). Since 2010, the National Action Plan for Roma Inclusion has focused on the promotion of education, employment, and social protection among Egyptian and Roma communities to promote their inclusion and equal opportunities in society (Home Office, 2017). Despite some achievements, some concerning issues persist. For instance, although there was a reduction in school drop-out, rates still remain the highest in the country, with 1 to 5% of Egyptian and Roma people who have completed secondary education versus 94.5% for the overall population in Albania (Home Office, 2017; Psacharopoulos, 2017). Noteworthy, the pandemic represented an additional risk factor for school drop-out among Egyptian and Roma adolescents who were still enrolled in school because of barriers in attending online classes due to lack of internet connection and space at home (Dervishi et al., 2021; Miconi et al., 2021a).

### 1.2. Risk behaviors and well-being among Egyptian and Roma youth

Risk behaviors are one of the most investigated outcomes among Roma youth (Dimitrova and Ferrer-Wreder, 2017), with multiple studies reporting concerning levels of violence (e.g., getting into fights, illegal activities) and substance use/abuse (e.g., alcohol and drugs; Kósa et al., 2007; Cook et al., 2013; Netzelmann et al., 2016; Dimitrova and Ferrer-Wreder, 2017; Molnar, 2021). It is important to acknowledge that historically both media and research on Roma people contributed to the promotion of the stereotypical image of Roma adolescents as problematic and delinquent youth (Hammarberg, 2008; Molnar, 2021). However, a recent systematic review of victimization and delinquency among Roma youth and adults in Europe reported that, besides being at higher risk of discrimination and victimization, findings about Roma youth’s alcohol and drug use compared to non-Roma youth were mixed (Molnar, 2021). For instance, some studies found lower rates of substance abuse among Roma, especially among girls (Kolarcik et al., 2014), whereas higher rates of self-reported alcohol use and illicit drug use were found among Roma adolescents in Hungary (Gerevich et al., 2010). These mixed findings may be linked to methodological and contextual differences across studies and countries/communities. When socio-economic factors and victimization experiences are taken into account, Roma youth do not report higher risk behaviors than their non-Roma peers in the Czech Republic (Vazsonyi et al., 2016), with the only exception of drug use that may be more culturally accepted among Roma than non-Roma communities in some countries (Molnar, 2021).

Furthermore, when social desirability is taken into account, Roma youth in Slovakia did not differ from their non-Roma peers in terms of self-reported delinquent behaviors (Kolarcik et al., 2014). Evidence about gender differences in risk behaviors is also mixed, with some studies showing boys at higher risk than girls (Kósa et al., 2007; Cook et al., 2013; Kolarcik et al., 2014; Miconi et al., 2021a; Molnar, 2021) and others showing no significant differences (Gerevich et al., 2010). Less is known about Egyptian youth, although national reports suggest Egyptian and Roma youth report similar experiences in the country (De Soto et al., 2005; Republic of Albania, 2015). Such findings were supported by Miconi et al.’s (2021a) study that highlighted the presence of violent behaviors, delinquency, gambling, and substance use among both Roma and Egyptian youth during the pandemic. Although extensive research suggests that youth who drop-out of school are at higher risk of behavioral problems (Egger et al., 2003; Chou et al., 2006; Nik Jaafar et al., 2013), research on Egyptian and Roma youth who dropped out of school is very scarce (Miconi et al., 2021a). This is surprising in light of the 50% school drop-out rate that has been reported among Roma communities in Albania (Home Office, 2017; Psacharopoulos, 2017). The exclusion of youth who dropped out of school can be attributed to the fact that most studies including Roma youth were school-based (Miconi et al., 2021a; Wiium and Kozina, 2021). This, however, represents an important limitation and bias of existing research, as very little is known about the risk behaviors and assets of the large number of youths who are not enrolled in school anymore.

Fewer studies have focused on positive outcomes of well-being in these populations (Cabrera and Leyendecker, 2017; Leman et al., 2017; Motti-Stefanidi and García Coll, 2018; Yıldırım et al., 2022). In a PYD perspective, the focus on positive outcomes is important to overcome negative representations of minorities and focus on their assets rather than just on their weaknesses. Overall, adolescents’ well-being was found to be associated with positive health and behavioral patterns that persisted throughout adulthood (Patton et al., 2011; Currie et al., 2012; Lim et al., 2017). However, significant gaps exist in our understanding of Egyptian and Roma youth’s well-being and mental health. Cook’s and colleagues’ systematic review of health and well-being among Roma populations suggest overall lower well-being and higher depressive symptoms among Roma compared to non-Roma populations in Europe (Cook et al., 2013). Findings on gender differences in well-being among adolescents suggest that girls generally report lower scores of well-being compared to boys (Marquez and Long, 2021), and the same picture has been confirmed among Albanian youth (Lebedeva et al., 2018; Vazsonyi et al., 2020). The few studies that investigated well-being specifically in Roma youth found overall low levels of well-being, but gender differences are yet unclear and in need of further research (Dimitrova and Ferrer-Wreder, 2017; Dimitrova et al., 2017b).

Little is known about how the situation of Egyptian and Roma youth has evolved during the pandemic. Preliminary findings suggest that the COVID-19 pandemic jeopardized both Egyptian and Roma adolescents’ well-being (Miconi et al., 2021a), increasing isolation and mental health concerns. Such findings pointed to a possible increase in risk behaviors among boys as a way to deal with the stress of the situation, and to an overall decrease of well-being and mental health especially among girls (Miconi et al., 2021a), mirroring the well-established literature on the predominance of internalizing problems in women (Vazsonyi et al., 2020) and of externalizing problems in men (Kósa et al., 2007; Cook et al., 2013; Kolarcik et al., 2014; Molnar, 2021). To the best of our knowledge no studies provided information on well-being and risk behaviors among Egyptian youth specifically, nor on Egyptian or Roma youth who dropped out of school. Nonetheless, high school drop-out has been associated with negative long-term difficulties, including unemployment, arrest and incarceration and substance abuse (Lansford et al., 2016), suggesting that school drop-out can significantly undermine youth’s positive development.

### 1.3. Personal and contextual assets

Based on a PYD perspective, developmental assets are described as essential resources that youth depend on to thrive in their current and future lives (e.g., academic and career success, healthy social relationships, and well-being) and include external/contextual assets and internal/personal assets (Benson, 2003). Specifically, contextual assets are resources that youth can access at school, in their family or in their community. Personal assets are individual resources in terms of personality and abilities that the youth will acquire over time. Although there has been some research exploring the validity and applicability of measures of developmental assets in low-and-middle-income countries, most studies to date have been conducted in the North American context. Much less is known on developmental assets among minority youth in the Eastern European context.

Empirical research suggests that the presence of developmental assets is associated with fewer risk behaviors regardless of gender, ethnicity, socioeconomic status, and geographical area (Scales, 2011; Syvertsen et al., 2021). The limited research conducted among Albanian adolescents suggests that Albanian youth have access to a moderate level of developmental assets (Scales, 2011; Nano, 2012). Specifically, Albanian youth reported counting on the support of the family and school contexts (Scales, 2011) and their positive values, social competencies and positive identities were negatively related to risk behaviors (Uka et al., 2021). Most research on youth’s developmental assets with Roma youth in Eastern Europe has been conducted in the school context (Dimitrova et al., 2014, 2017a,b, 2018; Wiium and Uka, 2021). Results of these studies indicate that the family is a major contextual asset for Roma youth across multiple countries in Europe, and represents a crucial contextual asset in everyday life as well as in situations of adversity and distress (Dimitrova et al., 2014).

During the pandemic, Miconi et al. (2021a) in their qualitative study highlighted how Egyptian and Roma adolescents in Albania reported overall low developmental assets, especially in terms of positive identity. Although still low, adolescents mentioned positive values and social competencies as protective for their development and well-being (Miconi et al., 2021a). Their results confirmed that family support represented a crucial developmental asset for youth. However, neighborhoods and schools were depicted at times as unsafe contexts at increased risk of discrimination, violence and victimization, with very few resources to offer to minority youth (Miconi et al., 2021a). Although Roma youth often live in segregated and unsafe neighborhoods (Kurtenbach, 2021), little research has focused on the association between neighborhood assets and Roma’s youth development. The available findings suggest that Roma adolescents’ neighborhoods are risk factors for both internalizing problems and risk behaviors, similarly for boys and girls (Vazsonyi et al., 2020). Hence, in the present study we focus on perceived family and neighborhood support in terms of contextual assets. Whether ethnic, gender or education differences between adolescents who are enrolled in school versus those who dropped out exist in levels of contextual and personal developmental assets and in the associations among such developmental assets, risk behaviors and well-being has yet to be determined.

### 1.4. The current study

We adopted a PYD and a collaborative, community-based approach as to be able to recruit Egyptian and Roma adolescents enrolled in school as well as those who had dropped out of school. The resilience-oriented approach as well as the community-based recruitment strategy are two strengths of the present study, both contributing to overcoming limitations of prior studies which were mostly school-based and focused exclusively on negative outcomes and risks among minority youth. In addition, the data collection took place during the first wave of the pandemic providing a unique context to assess risk behaviors, well-being, and developmental assets among these understudied and at-risk minority groups. Research on the experiences of minority youth during the pandemic is essential to inform prevention and intervention efforts able to mitigate the widening of health and social inequalities.

We investigated in a sample of Egyptian and Roma adolescents living in Albania during the first wave of the COVID-19 pandemic: (1) levels of self-reported risk behaviors, well-being, personal (e.g., positive identity, positive values, social competencies) and contextual (e.g., family, neighborhood) assets; (2) whether these variables differed by ethnicity, gender and education (enrolled in school vs. dropped-out of school); (3) associations of personal and contextual assets with risk behaviors and well-being; (4) whether these associations differed by ethnicity, gender, and education.

Overall, based on the very limited available research on Egyptian and Roma youth and their concerning situation in Albania and during the pandemic, we expected high levels of risk behaviors and low levels of well-being in our sample (Miconi et al., 2021a). We expected overall low levels of developmental assets, with higher scores reported for family support (Dimitrova et al., 2014; Miconi et al., 2021a). Notwithstanding the scarcity of data, given the very similar situation of Egyptian and Roma youth in Albania (De Soto et al., 2005; Republic of Albania, 2015), we did not expect ethnic differences in levels of reported assets, risk behaviors or well-being. In light of prior research on gender differences in mental health we would expect boys to report more risk behaviors and higher well-being than girls, and adolescents who dropped out of school to report fewer assets, more risk behaviors and lower well-being than their peers enrolled in school. We expected available assets to be positively associated with well-being and negatively associated with risk behaviors similarly across groups (Scales et al., 2017; Vazsonyi et al., 2020; Wiium and Kozina, 2021).

## 2. Methods

### 2.1. Participants

A total of 201 Egyptian and Roma adolescents (49.8% Roma; *M_age_* = 16.63, *SD_age_* = 1.80; 47% girls; 53% school dropouts) completed a series of questionnaires in the form of an individual interview with a trained research assistant in a community setting. Most adolescents were single (88.6%) and identified as Muslims (95%). Only 5% of adolescents identified as Catholic and 11.4% were married. In terms of educational level of their parents, 55.7% of adolescents reported that their parents had no education, 12.9% that one of their parents had completed at least primary school, and 31.3% that both parents had completed at least primary school (Table 1).

**Table 1:** Descriptive statistics of socio-demographic variables for the overall sample and separately by ethnic group, gender group and education group (*N* = 201).

Variables	Ethnicity				Gender			Education group		
	Total sample	Roma	Egyptian	*OR*	Boys	Girls	*OR*	No education/dropout	At school/Completed school	*OR*
	*N (%)*	*n (%)*	*n (%)*		*n (%)*	*n (%)*		*n (%)*	*n (%)*	
Marital status										
Single	178 (88.6)	82 (82.0)	96 (95.0)		100 (93.5)	78 (83.0)		87 (81.3)	96(96.8)	
Married	23 (11.4)	18 (18.0)	5 (5.0)	0.24^***^	7 (6.5)	16 (17.0)	2.93^*^	20 (18.7)	3 (3.2)	0.14^***^
Ethnicity										
Roma	100 (49.8)				55 (51.4)	45 (47.9)		79 (73.8)	21 (22.3)	
Egyptian	101 (50.2)				52 (48.6)	49 (52.1)	1.15	28 (26.2)	73 (77.7)	9.81^***^
Gender										
Boys	107 (53.2)	55 (55.0)	52 (51.5)					48 (44.9)	59 (62.8)	
Girls	94 (46.8)	45 (45.0)	49 (48.5)	1.15				59 (55.1)	35 (37.2)	0.48^*^
Education group										
No education/drop out	107 (53.2)	79 (79.0)	28 (27.7)		48 (44.9)	59 (62.8)				
At school/ completed school	94 (46.8)	21 (21.0)	73 (72.3)	9.81^***^	59 (55.1)	35 (37.2)	0.48^***^			
Parental education										
No education/drop out	112 (55.7)	79 (79.0)	33 (32.7)		54 (50.5)	58 (61.7)		92 (86.0)	20 (21.3)	
One parent completed at least primary education	26 (12.9)	17 (17.0)	9 (8.9)	1.27	18 (16.8)	8 (8.5)	0.41	10 (9.3)	16 (17.0)	7.36^***^
Both parents completed at least primary education	63 (31.3)	4 (4.0)	59 (58.4)	35.31^***^	35 (32.7)	28 (29.8)	0.74	5 (4.7)	58 (61.7)	53.36^***^
Age (Mean, SD)	16.6 (1.8)	16.7 (1.8)	16.6 (1.8)	*F*_*(1*,*199)*_ *=* 0.09, η_*p*_^2^ = 0.001	16.8 (1.6)	16.5 (2.0)	*F*_*(1*,*199)*_ *=* 1.31, η_*p*_^2^ = 0.007	16.5 (1.9)	16.8 (1.6)	*F*_*(1*,*199)*_ *=* 0.86, η_*p*_^2^ = 0.004

OR, odds ratio; SD, standard deviation; *p < 0.05; **p < 0.01; ***p < 0.001.

### 2.2. Procedure

A convenience sample of adolescents was recruited *via* the collaboration with four communities, non-profit organizations working with the Roma and Egyptian communities in different Albania regions. Recruitment was conducted *via* personal contacts within the organizations by cultural mediators known to the community. The first contact for participants under the age of 18 was made with the potential participants’ parents. If parents agreed to the participation of their child in the study, they were asked to sign a parental informed consent. After that, selected adolescents (minors) were contacted and asked for their interest in participation and verbal assent. Participants aged 18 years or older were asked directly for their interest and signed an informed consent. The community-based recruitment strategy is the main strength of the project, as it allowed us to include Egyptian and Roma adolescents who dropped out of school and who are excluded from most research and interventions in the country, especially during the pandemic. Adolescents completed a series of validated questionnaires in the form of an individual interview with a trained research assistant. Participants were given a gift of the value of two euros for their time. The study protocol and procedures were approved by the ethics committee of the University of Bergen (Norway) (Reference number: 612969).

### 2.3. Measures

#### 2.3.1. Dependent variables

##### 2.3.1.1. Risk behaviors

In line with Benson’s developmental assets framework, risk behaviors were measured with the risk behaviors scale of the Developmental Assets Profile developed by the Search Institute (Benson, 2007). Participants were asked to answer 13 questions with a yes/no response option (i.e., “Have you used alcohol once or more in the last 30 days?,” “Have you used other illicit drugs (e.g., cocaine, LSD, heroin, amphetamines, etc.) once or more in the last 12 months?,” “Have you been in a group fight once or more in the last 12 months?”). All yes responses were recoded as 1 and no responses were recoded as 0. A total score of risk behaviors was calculated by summing the responses to all items, with possible scores going from 0 to 13, and higher scores indicating more risk behaviors. Prior findings supported the validity of the risk behavior scale in a range of cultural settings (i.e., youth in Albania, Bangladesh, Japan, Lebanon, and the Philippines; Scales, 2011), as well as among minority students with different ethnic backgrounds in the US (Pashak et al., 2018).

##### 2.3.1.2. Well-being

Well-being was measured with the World Health Organization Well-being Index (WHO-5; Dadfar et al., 2018). The WHO-5 scale consists of 5 items rated on a 6-point Likert scale (0 = at no time; 5 = all the time). Participants are asked to indicate how often they experienced the situation described by each item over the past 2 weeks (i.e., “I have felt cheerful and in good spirits”). Higher scores indicate higher well-being. Validity of this scale has been confirmed among ethnic minority and majority adolescents (Sirpal et al., 2016). Cronbach’s alpha and Omega in our overall sample were both 0.98.

#### 2.3.2. Independent variables

##### 2.3.2.1. Developmental assets

Subscales of the Developmental Assets Profile (DAP) by the Search Institute (Benson, 2007) were used as a measure of youth’s developmental assets. The DAP consists of items assessing young people’s experience of developmental assets. They are divided into personal and contextual assets categories. The contextual assets include Support from family (seven items, e.g., “I have a family that gives me love and support”) and neighborhood (six items, e.g., “I have good neighbors who care about me”). The personal assets consist of Positive Values (seven items, e.g., “I am encouraged to help others”), Social Competencies (seven items, e.g., “I accept people who are different from me”), and Positive Identity (eight items, e.g., “I feel good about myself”). Responses were rated on a 4-point Likert scale (1 = not at all or rarely, 4 = extremely or almost always), with higher scores indicating a higher presence of assets. Cronbach’s alphas and Omegas in our overall sample for each subscale of the contextual assets were: *family assets* α = 0.66 and ω = 0.67; *neighborhood* assets α = 0.80 and ω =0.80. As for personal assets, Cronbach’s alphas and Omegas in our overall sample were: *social competence* α = 0.88 and ω = 0.89, *positive values* α = 0.77 and ω = 0.78, and *positive identity* α = 0.83 and ω = 0.79.

#### 2.3.3. Socio-demographic variables

Participants reported on their gender (i.e., boy or girl), age, ethnicity (i.e., Egyptian or Roma), religion (i.e., Muslim or Catholic), level of parental education (i.e., no education/at least one parent completed at least primary education/both parents completed at least primary education), marital status (i.e., single or married), and their education level (i.e., at school/completed school vs. dropped out of school).

### 2.4. Data analysis

Analyses were conducted using R software and RStudio (R Core Team, 2021). There were no missing data in our dataset. Descriptive information for the sample was summarized using means and standard deviations for continuous variables and counts and proportions for categorical variables, in the overall sample and separately by ethnicity, gender and education group (i.e., at school vs. dropped-out of school). At a descriptive level, we used ANOVA and logistic regression to examine univariable associations of students’ socio-demographic characteristics, risk behaviors, well-being and developmental assets with ethnicity, gender, and education group.

A methodological issue that often prevents quantitative researchers to conduct meaningful statistical analyses with minority youth is the difficulty to establish measurement invariance across groups of interest (Milfont and Fischer, 2010). In the present study, prior to conducting multivariable analyses we established the measurement invariance of all scales across groups (ethnic groups, gender groups, education groups) *via* multi-group Confirmatory Factor Analysis using the *lavaan* library (Rosseel, 2012) as to ensure the validity of our scales and of comparisons of means and associations across groups. The Diagonally Weighted Least Squares (DWLS) estimator for ordinal data was used. Most of the items in our measures showed a skewed distribution, further supporting the appropriateness of an analytical approach which takes the ordinal nature of the data into account (Flora and Curran, 2004). Given the marked asymmetric distribution of most item responses across scales, responses were recoded as a preliminary step to enable us to run a CFA (i.e., preliminary analyses suggested a two-point response format for all scales; for the well-being, family, social competencies, positive values and positive identity scales: Not at all/sometimes = 0, all other responses = 1; for the neighborhood scale: Not at all = 0, all other responses = 1). We followed a step-by-step procedure. First, models were fitted separately by ethnic group. Second, configural invariance was tested by allowing the parameters to remain free across groups (i.e., ethnic groups, gender groups and education groups, separately). Third, metric and scalar invariance were tested simultaneously by constraining the factor loadings and thresholds to be equal across groups (separately for ethnic groups, gender groups and education groups; Muthén and Muthén, 1998). Results were evaluated following the general guidelines suggested by Chen (2007). Specifically, several fit indexes (i.e., comparative fit index CFI, Tucker-Lewis index TLI and root mean square error of approximation RMSEA) were considered; values of RMSEA less than or equal to 0.06, and a CFI and TLI greater than 0.95 were considered to indicate a good fit of the model. The ΔCFI and ΔRMSEA were computed between the two proximal models (i.e., configural vs. metric and scalar). A difference in ΔCFI <0.01 and a difference in ΔRMSEA <0.015 were considered evidence for model invariance. Given the limited power of running a multi-group CFA in our relatively small sample, we respected the original validated metric of the questionnaires in all subsequent analyses (Moscardino et al., 2020).

Prevalence of risk behaviors, levels of well-being and developmental assets in the overall sample and across groups of interest were assessed *via* binomial and linear regression models. All models included gender, ethnic group and education group, and were adjusted for relevant socio-demographic variables (i.e., age and parents’ educational level). Next, the potential protective role of personal and contextual factors for well-being and risk behaviors were assessed *via* Poisson and linear regression models to assess the association between adolescents’ personal and contextual assets and our outcomes of interest (i.e., well-being and total risk behaviors scores). Variability in the potential protective role of individual and contextual factors for well-being and risk behaviors across ethnic groups, genders and education groups were assessed by including interaction terms for each asset with ethnicity, gender, and education groups in separate models, one at the time (for each outcome of interest).

## 3. Results

### 3.1. Preliminary analyses: Descriptive statistics about socio-demographic variables and multi-group CFA

Descriptive statistics in the overall sample and by groups of interest are reported in Tables 1, 2A,2B. Roma youth had higher levels of school drop-out, fewer parents who had completed their education, and were more likely of being married than their Egyptian peers. Boys were less likely of being married and of dropping out of school than girls. Adolescents who dropped out of school were more likely girls and married and reported a lower level of parental education (see Table 1).

**Table 2A:** Descriptive statistics of single risk behaviors for the overall sample and separately by ethnic group, gender group and education group (*N* = 201).

Variables		Total sample		Ethnicity					Gender				Education group					
				Roma		Egyptian			Boys		Girls			No education/drop -out		At school/completed school		
*N*	*%*	*n*	*%*	*n*	*%*	*OR*	*n*	*%*	*n*	*%*	*OR*	*n*	*%*	*n*	*%*	*OR*
Used alcohol once or more in the last 30 days	Yes	99	49.3	50	50	49	48.5	0.94	93	86.9	6	6.4	0.01^***^	44	41.1	55	58.5	2.02^*^
Drunk once or more in the last 30 days	Yes	48	23.9	30	30	18	17.8	0.51	44	41.1	4	4.3	0.06^***^	25	23.4	23	24.5	1.06
Smoked cigarette once or more in the last 30 days	Yes	103	51.2	54	54	49	48.5	0.8	87	81.3	16	17	0.05^***^	49	45.8	54	57.4	1.6
Sniffed or inhaled substances to get high once or more in the last 12 months	Yes	23	11.4	10	10	13	12.9	1.33	22	20.6	1	1.1	0.04^***^	9	8.4	14	14.9	1.91
Used other illicit drugs once or more in the last 12 months	Yes	13	6.5	7	7	6	5.9	0.84	12	11.2	1	1.1	0.09^***^	5	4.7	8	8.5	1.9
Engaged in sexual intercourse once or more in lifetime	Yes	55	27.4	31	31	24	23.8	0.69	34	31.8	21	22.3	0.62	31	29	24	25.5	0.84
Involved in shop lifting once or more in the last 12 months	Yes	93	46.3	46	46	47	46.5	1.02	62	57.9	31	33	0.36^***^	50	46.7	43	45.7	0.96
Committed vandalism once or more in the last 12 months	Yes	37	18.4	21	21	16	15.8	0.71	35	32.7	2	2.1	0.04^***^	20	18.7	17	18.1	0.96
In a group fight once or more in the last 12 months	Yes	111	55.2	58	58	53	52.5	0.8	68	63.6	43	45.7	0.48^*^	60	56.1	51	54.3	0.93
Carried a weapon for protection once or more in the last 12 months	Yes	6	3	5	5	1	1	0.19	5	4.7	1	1.1	0.22	5	4.7	1	1.1	0.22
Gambled with money once or more in the last 12 months	Yes	71	35.3	27	27	44	43.6	2.09^*^	64	59.8	7	7.4	0.05^***^	28	26.2	43	45.7	2.38^**^
Have felt sad most or all of the time without cause in the last month	Yes	80	39.8	31	31	49	48.5	2.1^*^	46	43	34	36.2	0.75	38	35.5	42	44.7	1.47
Attempted suicide one or more times	Yes	18	9	17	17	1	1	0.05^***^	9	8.4	9	9.6	1.15	14	13.1	4	4.3	0.3^*^

OR, odds ratio; *p < 0.05; **p < 0.01; ***p < 0.001.

**Table 2B:** Descriptive statistics of total risk behaviors, developmental assets and well-being for the overall sample and separately by ethnic group, gender group and education group (*N* = 201).

Variables	Total sample		Ethnicity						Gender						Education group						Roma		Egyptian				Boys		Girls				No education/drop-out		At school/Completed school				*M*	*SD*	*M*	*SD*	*M*	*SD*	*F*_*(1*,*199)*_	η_*p*_^2^	*M*	*SD*	*M*	*SD*	*F*_*(1*,*199)*_	η_*p*_^2^	*M*	*SD*	*M*	*SD*	*F*_*(1*,*199)*_	η_*p*_^2^
Risk behavior (total sum)	3.77	2.62	3.87	2.34	3.66	2.87	0.312	0.002	5.43	2.22	1.87	1.51	171.08^***^	0.462	3.53	2.51	4.03	2.72	1.83	0.009
Family assets	2.37	0.42	2.35	0.46	2.41	0.37	0.802	0.004	2.22	0.41	2.55	0.37	35.12^***^	0.150	2.34	0.47	2.41	0.35	1.69	0.008
Neighborhood assets	1.66	0.39	1.67	0.43	1.65	0.35	0.035	<0.001	1.66	0.38	1.66	0.41	0.01	<0.001	1.63	0.42	1.69	0.34	1.07	0.005
Positive values	2.31	0.41	2.27	0.43	2.32	0.39	0.918	0.005	2.21	0.41	2.39	0.39	10.47^***^	0.050	2.28	0.43	2.31	0.39	0.24	0.001
Positive identity	2.04	0.46	2.01	0.44	2.08	0.47	1.502	0.008	2.01	0.48	2.07	0.43	0.76	0.004	1.96	0.41	2.14	0.48	8.20^***^	0.039
Social competencies	2.38	0.48	2.34	0.51	2.42	0.46	1.607	0.008	2.31	0.52	2.46	0.44	4.38^***^	0.022	2.29	0.48	2.48	0.48	7.70^***^	0.037
Well-being	2.47	0.68	2.39	0.65	2.55	0.71	2.607	0.03	2.46	0.69	2.49	0.67	0.09	<0.001	2.41	0.72	2.55	0.62	2.68	0.013

***p < 0.001.

Results of multi-group CFA across ethnic groups, gender groups and education groups are reported in Tables 3–5, respectively. Scalar invariance was established across ethnic groups for all scales. As regards gender groups and education groups, scalar invariance was established for all scales but the positive identity scale. Hence, score differences across gender and education groups on the positive identity scale should be interpreted with caution.

**Table 3:** Results of multi-group CFAs by ethnic group (*N* = 201).

Variables	Chi-square test statistics	*p*	CFI	∆CFI	TLI	∆TLI	RMSEA	∆RMSEA
Well -being
Configural invariance	1.58	0.999	0.999		0.998		0.001	
Metric and scalar invariance	3.05	0.999	0.998	0.001	0.997	0.001	0.002	0.001
Family assets
Configural invariance	14.72	0.981	1.000		1.008		0.001	
Metric and Scalar invariance	30.48	0.593	0.999	0.001	1.001	0.007	0.002	0.001
Neighborhood assets
Configural invariance	33.14	0.411	1.000		1.000		0.019	
Metric and scalar invariance	40.43	0.369	0.999	0.001	0.999	0.001	0.025	0.006
Social competencies
Configural invariance	14.41	0.999	0.999		0.998		0.001	
Metric and scalar invariance	24.54	0.974	0.998	0.001	0.997	0.001	0.003	0.002
Positive values
Configural invariance	27.24	0.938	0.999		0.998		0.001	
Metric and scalar invariance	37.99	0.823	0.998	0.001	0.997	0.001	0.002	0.001
Positive identity
Configural invariance	10.17	0.425	0.999		0.998		0.013	
Metric and scalar invariance	14.93	0.456	0.998	0.001	0.997	0.001	0.001	0.012

Due to collinearity issues, three residual co-variances were included in the social competencies model, four in the neighborhood assets model, seven in the positive values model and nine in the positive identity model.

**Table 4:** Results of multi-group CFAs by gender group (*N* = 201).

Scales	Chi-square test statistics	*p*	CFI	∆CFI	TLI	∆TLI	RMSEA	∆RMSEA
**Well-being**
Configural invariance	1.58	0.992	1.000		1.000		0.001	
Metric and scalar invariance	1.71	0.990	0.999	0.001	0.999	0.001	0.002	0.001
**Family assets**
Configural invariance	14.56	0.983	1.000		1.014		0.001	
Metric and scalar invariance	18.87	0.977	0.999	0.001	1.013	0.001	0.002	0.001
**Neighborhood assets**
Configural invariance	21.1	0.929	1.000		1.004		0.001	
Metric and scalar invariance	22.99	0.974	0.999	0.001	1.003	0.001	0.002	0.001
**Social competencies**
Configural invariance	14.568	0.999	1.000		1.000		0.001	
Metric and scalar invariance	18.909	0.998	0.999	0.001	0.999	0.001	0.002	0.001
**Positive values**
Configural invariance	22.58	0.988	1.000		1.004		0.001	
Metric and Scalar invariance	38.19	0.817	0.999	0.001	1.002	0.002	0.002	0.001
**Positive identity**
Configural invariance	15.32	0.121	1.000		0.998		0.073	
Metric and scalar invariance	18.72	0.226	0.999	0.001	0.999	0.001	0.050	0.023

**Table 5:** Results of multi-group CFAs by education group (*N* = 201).

Scales	Chi-square test statistics	*p*	CFI	∆CFI	TLI	∆TLI	RMSEA	∆RMSEA
**Well-being**
Configural invariance	1.82	0.998	1.000		1.000		0.001	
Metric and scalar invariance	2.62	0.990	0.999	0.001	0.999	0.001	0.002	0.001
**Family assets**
Configural invariance	11.67	0.997	1.000		1.014		0.001	
Metric and scalar invariance	26.79	0.769	0.999	0.001	1.005	0.009	0.002	0.001
**Neighborhood assets**
Configural invariance	27.51	0.693	1.000		1.001		0.001	
Metric and scalar invariance	32.04	0.740	0.999	0.001	0.999	0.002	0.002	0.001
**Social competencies**
Configural invariance	12.294	0.999	1.000		1.000		0.001	
Metric and scalar invariance	15.896	0.999	0.999	0.001	0.999	0.001	0.002	0.001
**Positive values**
Configural invariance	47.42	0.196	1.000		0.999		0.043	
Metric and scalar invariance	55.41	0.187	0.999	0.001	0.998	0.001	0.042	0.001
**Positive identity**
Configural invariance	14.29	0.163	11.000		1.000		0.065	
Metric and scalar invariance	18.13	0.256	0.999	0.001	0.999	0.001	0.046	0.019

### 3.2. Self-reported risk behaviors, well-being and developmental assets

Around half of participants reported having drunk alcohol once or more in the last 30 days, smoking cigarettes or getting in a group fight, these three being the most common risk behaviors reported by adolescents in our sample. Another 46.3% of adolescents reported being involved in shop lifting once or more in the past year and 39.8% reported feeling sad most or all the time without a cause in the past month (see Tables 2A,2B).

Overall, participants reported average levels of well-being (*M* = 2.47, *SD* = 0.68, range: 0–5) and low scores across all developmental assets (range: 1–4). Specifically, the highest scores were reported for family assets (*M* = 2.37, *SD* = 0.42), whereas the lowest scores were reported for positive identity (*M* = 2.04, *SD* = 0.46) and neighborhood assets (*M* = 1.66, *SD* = 0.39).

### 3.3. Group differences in well-being, risk behaviors and developmental assets

In multivariable statistical analyses, no statistically significant differences emerged across groups in terms of self-reported well-being (Table 6). Only one significant difference emerged for total of risk behaviors, with girls reporting overall fewer risk behaviors than boys (Table 7). Specifically, when looking at risk behaviors (Table 8), girls were less likely of presenting most of the assessed risk behaviors, with the exception of sex intercourses, carrying weapons, reporting feeling sad without a reason and suicide attempts for which no gender differences emerged. However, very few adolescents reported carrying a weapon overall (six adolescents). Egyptian youths were more likely of gambling money and feeling sad without a cause, but less likely of reporting suicide attempts compared to their Roma peers. No significant differences in risk behaviors emerged between adolescents who had completed secondary school or who were still enrolled in school and those who dropped out of school (see Tables 7, 8).

**Table 6:** Results of linear regression models on well-being and developmental assets looking at the main effects of ethnicity, gender and education group (*N* = 201).

	Predictors									Ethnicity			Gender			Education group		
Outcome	*F*_1,199_	*p*	η_*p*_^2^	*F*_1,199_	*p*	η_*p*_^2^	*F*_1,199_	*p*	η_*p*_^2^
Well-being	0.004	0.982	0.0001	0.116	0.733	0.0006	0.003	0.950	0.0001
Family assets	0.384	0.541	0.0019	41.15	0.001	0.175	2.34	0.131	0.0118
Neighborhood assets	0.518	0.472	0.0026	0.173	0.677	0.0008	0.35	0.563	0.0017
Social competencies	0.402	0.532	0.002	7.482	0.006	0.0371	6.06	0.012	0.0302
Positive values	0.011	0.921	0.0005	10.33	0.001	0.051	0.02	0.883	0.0011
Positive identity	0.519	0.472	0.0026	2.329	0.128	0.0118	2.25	0.144	0.0114

All models controlled for participants’ age and level of parental education. Gender was coded 0 = boys and 1 = girls. Ethnicity was coded 0 = Roma and 1 = Egyptian. Education group was coded 0 = no education/drop-out of school and 1 = at school/completed school.

**Table 7:** Results of the Poisson regression model on total risk behaviors looking at the main effects of ethnicity, gender, and education group (*N* = 201).

Predictors		Dependent variable					Total risk behaviors (sum score)				
		AIRR	Robust SE	*t*-statistic	95% CI	*p*
Ethnicity	Roma	Ref				
	Egyptian	1.03	0.083	0.336	[0.88, 1.20]	0.069
Gender	Boys	Ref				
	Girls	0.34	0.032	−12.02	[0.29, 0.42]	0.001
Education group	No education/drop out	Ref				
	At school/completed school	1.02	0.085	0.192	[0.87, 1.20]	0.089

The model controlled for participants’ age and level of parental education; AIRR, adjusted Incidence rate ratio; SE, standard error; CI, Confidence interval; *p*, *p* value.

**Table 8:** Results of logistic regression models on specific risk behaviors looking at the main effects of ethnicity, gender and education group (*N* = 201).

Predictors			Ethnicity		Gender		Education group	
			Roma	Egyptian	Boys	Girls	No education/drop out	At school/completed school
**Outcome**	Used alcohol once or more in the last 30 days	AOR	1	1.42	1	0.01	1	2.05
		95% CI		[0.41, 5.26]		[0.00, 0.03]		[0.57, 7.93]
		*p*		0.586		0.001		0.284
	Drunk once or more in the last 30 days	AOR	1	0.5	1	0.06	1	0.92
		95% CI		[0.18, 1.31]		[0.02, 0.16]		[0.35, 2.41]
		*p*		0.169		0.001		0.871
	Smoked cigarette once or more in the last 30 days	AOR	1	0.96	1	0.05	1	1.54
		95% CI		[0.37, 2.50]		[0.02, 0.10]		[0.54, 4.60]
		*p*		0.936		0.001		0.424
	Sniffed or inhaled substances to get high once or more in the last 12 months	AOR	1	1.26	1	0.04	1	1.42
		95% CI		[0.35, 4.25]		[0.01, 0.20]		[0.42, 4.77]
		*p*		0.709		0.002		0.568
	Used other illicit drugs once or more in the last 12 months	AOR	1	0.81	1	0.11	1	1.41
		95% CI		[0.15, 3.61]		[0.01, 0.54]		[0.30, 6.37]
		*p*		0.788		0.031		0.664
	Engaged in sexual intercourse once or more in lifetime	AOR	1	0.56	1	0.59	1	0.64
		95% CI		[0.22, 1.38]		[0.29, 1.18]		[0.23, 1.67]
		*p*		0.217		0.138		0.369
	Involved in shop lifting once or more in the last 12 months	AOR	1	1.92	1	0.32	1	1.09
		95% CI		[0.90, 4.25]		[0.17, 0.60]		[0.47, 2.54]
		*p*		0.098		0.001		0.848
	Committed vandalism once or more in the last 12 months	AOR	1	1.03	1	0.04	1	0.92
		95% CI		[0.37, 2.85]		[0.01, 0.14]		[0.33, 2.55]
		*p*		0.952		0.001		0.879
	In a group fight once or more in the last 12 months	AOR	1	0.81	1	0.47	1	0.79
		95% CI		[0.39, 1.69]		[0.26, 0.85]		[0.35, 1.80]
		*p*		0.58		0.012		0.578
	Carried a weapon for protection once or more in the last 12 months	AOR	1	0.23	1	0.19	1	0.19
		95% CI		[0.01, 2.56]		[0.01, 1.24]		[0.01, 1.93]
		*p*		0.317		0.134		0.222
	Gambled with money once or more in the last 12 months	AOR	1	3.51	1	0.04	1	1.05
		95% CI		[1.32, 10]		[0.01, 0.09]		[0.40, 2.70]
		*p*		0.014		0.001		0.927
	Have felt sad most or all of the time without cause in the last month	AOR	1	2.58	1	0.73	1	1.12
		95% CI		[1.23, 5.50]		[0.40, 1.33]		[0.49, 2.56]
		*p*		0.013		0.301		0.782
	Attempted suicide one or more times	AOR	1	0.06	1	1.33	1	1.02
		95% CI		[0.00, 0.41]		[0.45, 4.08]		[0.21, 3.99]
		*p*		0.018		0.611		0.995

All models controlled for participants’ age and level of parental education. AOR, adjusted odds ratio; CI, confidence interval; p = *p* value; gender was coded 0 = boys and 1 = girls; ethnicity was coded 0 = Roma and 1 = Egyptian. Education group was coded 0 = no education/drop-out of school and 1 = at school/completed school.

In terms of developmental assets, girls reported more family assets, positive values and social competencies than boys. Youth who completed or who were still enrolled in school reported higher social competencies than their peers who had dropped out of school. No other differences across groups emerged neither for personal nor for contextual assets (see Table 6).

### 3.4. Associations of personal and contextual assets with risk behaviors and well-being

We found a positive significant association between positive identity and self-reported well-being (Table 9). Personal and contextual assets were not significantly associated with self-reported risk behaviors (Table 10).

**Table 9:** Results of the linear regression model on well-being looking at the main effects of developmental assets (*N* = 201).

Predictors	Dependent variable						
Developmental assets	Well-being						
	Estimate	SE	*T*-statistic	95% CI	*F*_*1*,*199*_	*p*	η_*p*_^2^
Family total	0.045	0.13	0.348	[−0.2, 0.30]	0.121	0.728	0.0006
Neighborhood total	−0.048	0.13	−0.380	[−0.31, 0.21]	0.144	0.704	0.0007
Social competencies	−0.044	0.16	−0.270	[−0.37, 0.28]	0.074	0.785	0.0003
Positive values	−0.007	0.18	−0.040	[−0.36, 0.34]	0.001	0.968	0.0001
Positive identity	0.387	0.13	2.923	[0.13, 0.67]	8.544	0.003	0.0432

The model included participants’ age, level of parental education, ethnicity, gender, and education group. SE, standard error; CI, confidence interval; *p*, *p* value.

**Table 10:** Results of the Poisson regression model on total risk behaviors looking at the main effects of developmental assets (*N* = 201).

Predictors	Dependent variable				
Developmental assets	Total risk behaviors (sum score)				
	AIRR	Robust SE	*T*-statistic	95% CI	*p*
Family total	1.12	0.092	1.090	[0.95, 1.31]	0.177
Neighborhood total	0.98	0.075	−0.226	[0.84, 1.14]	0.770
Social competencies	1.20	0.141	1.468	[0.95, 1.50]	0.124
Positive values	0.91	0.111	−0.754	[0.71, 1.15]	0.419
Positive identity	0.97	0.105	−0.275	[0.78, 1.20]	0.791

The model included participants’ age, level of parental education, ethnicity, gender, and education group. AIRR, adjusted incidence rate ratio; SE, standard error; CI, confidence interval; p, *p* value.

Interaction analyses yielded two significant interactions: the association between positive identity and well-being differed between Roma and Egyptian adolescents, with a higher association reported among Egyptian youth (*Β* = 0.57; *SE* = 0.23; *p* = 0.013). The association between family assets and risk behaviors also differed across ethnic groups, with family assets being more protective among Egyptian youth than among Roma youth (*AIRR* = 0.65; *SE* = 0.19; *p* = 0.022). No other significant results emerged from interaction analyses, suggesting that associations between most assets and our outcomes of interest did not vary by ethnic group, gender, or education group (see Supplemental Material).

## 4. Discussion

Although mounting evidence denounces how the pandemic is exacerbating social and health disparities in our society (Miconi et al., 2021b; Venkatesh et al., 2021) and impacting negatively especially adolescents’ development and mental health (Loades et al., 2020; Nearchou et al., 2020), research on vulnerable minority youth within a PYD approach during the pandemic is still scarce. This is even more true for Egyptian and Roma minority youth in Albania, two understudied communities who have endured a long history of oppression and marginalization in the country (De Soto et al., 2005; Republic of Albania, 2015) and who are likely to be particularly affected by the negative short- and long-term consequences of COVID-19 on psycho-social adjustment and well-being (Benner and Mistry, 2020; Larkins, 2020; Hawes et al., 2021; Miconi et al., 2021a). This community-based quantitative study adopted a PYD approach to assess levels of risk behaviors and well-being among a sample of Egyptian and Roma minority youth in Albania during the first wave of the COVID-19 pandemic. We investigated self-reported personal and contextual assets and their associations with risk behaviors and well-being. Our community-based recruitment strategy allowed us to include adolescents who had dropped out of school and who have been mostly excluded from prior research; in addition, the establishment of scalar measurement invariance across scales allowed us to investigate differences in risk-behaviors, well-being and developmental assets across ethnic groups, genders and education groups (adolescents who dropped out of school vs. adolescents who completed secondary school or who are still attending school).

### 4.1. Socio-demographic portrait

The socio-demographic characteristics of our sample confirmed the very low education level in these communities, with over half of the adolescent sample confirming that both their parents had no education or did not complete primary school (Miconi et al., 2021a; Uka et al., 2021). Albeit being low in both communities, the level of parental education reported was lower among Roma youth than among Egyptian youth. Adolescents who came from families with a lower educational level were more likely to drop out of school. As regards adolescents’ school drop-out, it seemed to be more of a problem among Roma than among Egyptian adolescents, among girls than among boys, and among married than single participants. Roma adolescents were more likely to be married than Egyptian youth. These results suggest that Roma youth may encounter more barriers to accessing education than their Egyptian peers; this finding underlines the importance of interpreting risks within an intersectionality approach (Orton et al., 2019).

Low level of education in the family, early marriages and financial difficulties may represent some important barriers to school attendance and success in these communities (Lever, 2012; Levinson, 2015; Helakorpi et al., 2020; Matras et al., 2020; Miconi et al., 2021a; Molnar, 2021). Early marriages among Roma communities remain a common practice and have been associated with multiple cultural and socio-economic factors; early marriage has been described as a “survival” strategy for girls in the community who are marginalized in the mainstream society both as women and members of an ethnic minority (Martsenyuk, 2015). Roma youth in Albania have mentioned that girls sometimes marry early as marriage is the easiest way for families to protect their children and keep them safe from the risks and contextual violence in their neighborhoods and society (Miconi et al., 2021a). In addition, rules of endogamy apply when it comes to marriages within the community, and rare disorders and private founder mutations can also be responsible of health issues associated with academic difficulties (Kalaydjieva et al., 2001). Although traditional gender roles are being reconsidered in many Roma communities, in order to allow Roma girls to stay in school, it is crucial to foster safety in those settings and improve the socio-economic conditions of Roma families at the societal level.

### 4.2. Self-reported risk behaviors, well-being and developmental assets

Our results highlighted concerning levels of risk behaviors, as well as low to average self-reported well-being and developmental assets during the pandemic, supporting our hypotheses. Whereas alcohol use and cigarette smoking can be common behaviors among youth in Albania in general, shop lifting, and group fights were also found to be common for around half of our sample. Around 18% of adolescents reported having used other drugs than alcohol or cigarettes in the past year. These levels of risk behaviors should be contextualized in light of the very difficult contexts of life of these youth in Albania as well as the lack of available assets. Such risk behaviors have been mentioned as ways to cope with the stress of their daily lives as well as the marginalization and violence in their contexts of life (Miconi et al., 2021a).

Egyptian and Roma communities face many structural barriers in the Albanian society, such as poverty, high school drop-out rates, unemployment, discrimination and marginalization (De Soto et al., 2005; Republic of Albania, 2015). These findings confirm results of a recent mixed-method study on a small sample of Egyptian and Roma youth in Albania during the pandemic (Miconi et al., 2021a), which showed gambling, substance use and group fights were common risk behaviors linked to the poor and unsafe neighborhoods where the youth lived. Noteworthy, 46% of adolescents reported feeling sad most of the time or all the time without a reason. This result confirms prior evidence showing how adolescents report very high levels of psychological distress during this pandemic (Loades et al., 2020; Nearchou et al., 2020; Miconi et al., 2021a). Recent studies have underlined how Roma youth do not differ from majority youth in levels of risk behaviors once their socio-demographic situation is accounted for. In light of the concerning economic situation in Albania, it is possible that similar levels of risk behaviors are common to youth from other minority or majority groups who are also experiencing important economic difficulties in the country. Nonetheless, these rates suggest that risk behaviors are common among youth from these communities and should be addressed by prevention and intervention programs aimed especially at improving the neighborhood conditions and safety of their contexts of life (Vazsonyi et al., 2020).

With regards to developmental contextual assets, our hypotheses were also confirmed. Despite being low, adolescents reported overall low to average family assets but very low neighborhood assets. This confirms prior findings on the importance of family among both of these communities (Scales, 2011; Wiium and Dimitrova, 2019), as well as the poverty and lack of resources that characterize the neighborhoods where these communities normally live (De Soto et al., 2005; Republic of Albania, 2015). Personal assets were all very low, with positive identity – which refers to one’s vision of the self and of the future – being the lowest. The low internal resources reported by participants may be interpreted in light of the long history of oppression of these communities in Albania, in that adolescents may have internalized the negative vision of themselves promoted by the “oppressors” (Prilleltensky and Gonick, 1996; Freire, 2017). It is also possible that the dire economic situation in Albania especially for young people (e.g., very high emigration and youth unemployment rates) may have contributed to the overall low levels of reported and perceived personal assets (Bosakova et al., 2020; Egi, 2020).

### 4.3. Group differences in well-being, risk behaviors and developmental assets

Egyptian and Roma youth reported similar levels of risk behaviors, well-being and developmental assets, confirming our expectations based on findings from the few available reports that suggest that these ethnic communities share a very similar and concerning situation in the country (De Soto et al., 2005). When looking closer at specific risk behaviors, gambling and sadness seemed to be a higher concern among Egyptian youth, whereas suicide attempts were more common among Roma adolescents. Despite their commonalities, these ethnic groups may present some specificities linked to their cultural and historical differences as well as to their contexts of life. More research among both communities is warranted to better understand ethnic similarities and differences in risk behaviors as well as the role that such risk behaviors may have for their coping and survival.

With regards to gender, our hypotheses were partially confirmed, as boys reported overall more risk behaviors than girls but did not differ in reported levels of well-being. The fewer risk behaviors reported overall by girls confirm prior findings on the higher prevalence of risk behaviors and externalizing problems among boys than girls (Kósa et al., 2007; Cook et al., 2013; Kolarcik et al., 2014; Vazsonyi et al., 2020; Molnar, 2021). Noteworthy, when looking at specific risk behaviors, all externalizing and criminalized activities were more frequently reported by boys than girls (e.g., substance use, fights, shop lifting). Yet, it is important to underline that some risk behaviors were still present and concerning among girls (i.e., shop lifting, getting into fights), suggesting the importance to take these risk behaviors into account in intervention programs targeting both genders in these communities. However, no gender differences emerged in internalizing risk behaviors (i.e., sadness and suicidal attempts) nor in engagement in sexual intercourses. This suggests that levels of psychological distress are similar for boys and girls in these communities, in contrast with most literature suggesting that boys generally report fewer internalizing difficulties than girls (Kósa et al., 2007; Cook et al., 2013; Kolarcik et al., 2014; Molnar, 2021). Whether this lack of difference indicates a real deviation from the expected gender differences rather than a better adaptation of girls and/or a worse adaptation of boys in these communities has yet to be determined. The similar engagement in sexual intercourses reported by boys and girls can be interpreted in light of the frequent early marriages that are common in these communities (Alvarez-Roldan et al., 2018; Orton et al., 2019; Molnar, 2021).

Girls reported more family assets, social competencies and positive values than boys. The higher perception of family assets among girls may be related to the clear expectations about women as caretakers in the family, which may provide girls with higher participation and connection to their family (Martsenyuk, 2015; Miconi et al., 2021a). Similarly, higher social competencies and positive values have been previously found among girls compared to boys, in line with prior studies reporting more prosocial assets in girls compared to boys across countries (Gomez-Baya et al., 2021; Wiium and Kozina, 2021).

Surprisingly and in contrast to our hypothesis, adolescents who dropped out of school did not report lower well-being or more risk behaviors than youth who were still attending school or who completed high school. Similarly, adolescents in school or those who completed school did not differ from their peers who dropped out of school in most of the reported developmental assets. This contradicts most of evidence that suggests that school represents an important contextual asset and protective factor in the lives of at-risk youth (Netzelmann et al., 2016; Dost-Gözkan et al., 2021). These counterintuitive findings may be related to the high levels of structural and direct violence and discrimination experienced by Egyptian and Roma youth in their neighborhoods as well as at school and in the society at large. Indeed, Miconi et al. (2021a) reported that discrimination from peers and teachers were common experiences among Egyptian and Roma youth in Albania, and that schools were often perceived as unsafe and violent places. The segregation of Roma and Egyptian children and adolescents in schools is common in Albania and represents a timely and hot political issue in the country (Dervishi et al., 2021; Likmeta, 2022; Taylor, 2022). It is urgent that the structural violence, segregation and marginalization of Roma and Egyptian communities in Albania and in Europe are acknowledged and addressed by prevention and intervention efforts. A community-based approach inspired by social justice principles is needed to protect and empower these communities; systemic institutional changes are a condition *sine qua non* to foster change, agency and well-being in these marginalized communities (Fésüs et al., 2012). Nonetheless, the higher social competencies reported by adolescents enrolled in school compared to their peers who dropped out is a promising finding that suggest that the school context can play a role in enhancing socio-emotional learning and further underlines the importance of interventions aimed to create safer environments for children and adolescents to allow youth to safely attend school. Indeed, cultural integration, the presence of cultural mediators from the community as well as the engagement of families and community members in school life have been found effective strategies to prevent school drop-out among Roma youth (García-Carrión et al., 2018).

### 4.4. Associations of personal and contextual assets with risk behaviors and well-being

With regards to the associations of personal and contextual assets with risk behaviors and well-being, our hypotheses were only partially supported. A higher positive identity was associated with higher well-being, confirming the important role of a positive vision of the self and the future for Roma and Egyptian adolescents (Miconi et al., 2021a). In addition, one’s positive identity seemed to be more protective for Egyptian than for Roma youth. However, positive identity scores were the lowest asset reported in our sample, which makes sense in light of the dire situation of youth in Albania as well as the marginalization and oppression of these minority groups in the country (King and Vullnetari, 2003). Moreover, the pandemic may have jeopardized even more Egyptian and Roma youth’s positive identity, reducing their opportunities and widening inequalities (Arslan and Yıldırım, 2021; Miconi et al., 2021a). However, the positive identity scale did not reach scalar invariance across all groups, and scores were very low in our sample, therefore our findings should be interpreted with caution.

In contrast to our expectations, no significant associations emerged between developmental assets and risk behaviors. One significant interaction suggested that family assets may be significantly associated with lower risk behaviors especially among Egyptian youth. Adopting an intersectionality perspective (Marks et al., 2020), this finding may be interpreted in light of the overall higher resources and educational level reported by Egyptian youth compared to Roma youth in our sample. Nonetheless, it is important to mention that family has consistently emerged across studies as a crucial developmental context and source of support for Roma youth (Dimitrova et al., 2014; Miconi et al., 2021a). The weak associations that we found overall in our sample may be related to the limited sample size, as well as to the very low levels of assets that were reported by participants, reducing the variance in our models.

Last, no other differences across ethnic, gender or education groups emerged in the associations between most developmental assets, well-being and risk behaviors, confirming prior findings that showed similar protective/risk factors for externalizing and internalizing behaviors among Roma and non-Roma youth (Vazsonyi et al., 2020).

### 4.5. Limitations

The study has several limitations that need to be acknowledged when interpreting the results. First, the cross-sectional design prevents us from drawing conclusions about causality. Longitudinal studies are needed to better understand the directionality of associations between developmental assets, well-being and risk behaviors among Egyptian and Roma adolescents. Second, we relied on a small convenience sample and results cannot be generalized to Egyptian or Roma youth living in other countries or communities in Albania. Future studies drawing on larger samples are needed to replicate and expand on our findings. Third, our study was entirely based on adolescents’ self-reports. Hence, social desirability and mono-method biases cannot be excluded. Future studies should rely on multiple informants and sources of information. Fourth, although measurement invariance could be established across groups, responses to the questionnaires were very skewed in our sample. This raises some questions on the appropriateness of using these scales with samples of vulnerable minority youth. Last, we decided to focus on two of the most stigmatized and discriminated minority groups in Albania based on our collaborations with community-organizations serving these communities. However, it is important to acknowledge that other minority or majority groups who share a similar concerning socio-economic situation in Albania have not been included in our research. Given the difficult economic situation in Albania and high youth unemployment rates, youth who struggle economically in Albania, either from minority or majority groups, may share some of the vulnerabilities reported by the Egyptian and Roma adolescents in our study. Future studies are needed to shed light on the specificities of majority and minority groups in the country.

### 4.6. Implications of findings

Intervention and prevention efforts with Egyptian and Roma youth are urgently needed during and in the aftermath of this pandemic as to mitigate its negative impact on the development and mental health of these communities and to prevent the widening of social and health inequalities between minority and majority youth in our societies. Of importance, our data were collected at the beginning of the pandemic, and the already difficult situation reported by youth then may have worsened even more over time. Comprehensive interventions that tackle the stigmatization and violence in the neighborhoods where these communities live are warranted. Community leaders and policy makers should prioritize the provision of safer neighborhoods and healthier and safer daytime and nighttime activities for adolescents. Similarly, schools need to provide equal educational access and support to Egyptian and Roma youth, as well as a safe environment for youth to develop and thrive. The systemic discrimination, marginalization and stigmatization of these communities in Albania is a long-standing reality that needs to be urgently addressed. The non-protective role of the school environment that emerged from our study is a concerning result that points to the deleterious effects of this structural violence for youth in the communities and underlines the importance for policy makers to improve the access to education as well as the quality of the education and experiences of Egyptian and Roma youth in the school context. To do so, student-centered learning strategies and the promotion of cultural integration *via* the presence of cultural mediators from the minority communities as well as the engagement of families and community members in the school decision-making processes were all found to be promising practices with Roma youth to prevent school drop-out (Fésüs et al., 2012).

Service providers should consider that risk behaviors are common among Egyptian and Roma boys and girls, with some specificities. We need to acknowledge that risk behaviors can represent for young people a survival strategy to cope with the distress faced in their difficult daily lives. Although empowering and supporting minority youth personal assets is necessary, we must at the same time address their contextual needs and provide these youth with the contextual resources they need, starting from safer environments. The alignment between personal and contextual assets among these minority youth is a challenge but should be an urgent priority for policy makers. A greater involvement of community members in the design, implementation and evaluation of interventions and policy initiatives is necessary to ensure the effectiveness of such interventions (Fésüs et al., 2012; Netzelmann et al., 2016).

Supporting these youths’ positive identities can be an effective strategy to orient their actions towards non-violent activities and contributions to society, provided they are given alternative resources to thrive and speak their voice in their contexts of life. Anti-oppressive community-based bottom-up interventions that actually involve young people from the community represent promising initiatives in this direction. Despite a similar and concerning situation shared by all adolescents, potential gender and ethnic differences should be considered in interventions and further investigated in future studies.

### 4.7. Conclusion

Despite its limitations, our study offers some important insights on ways to support Egyptian and Roma youth’s positive development during and in the aftermath of this health emergency. Our community-based recruitment strategy allowed us to include in the study the unique and underrepresented perspectives of Egyptian and Roma minority adolescents who dropped out of school and that are often excluded from research and intervention initiatives. Policy makers, schools and community organizations should make more efforts to offer safe environments where minority youth can thrive, as well as empower them and actively involve them in research and intervention initiatives. The structural violence experienced by Egyptian and Roma youth at the societal level as well as in most of their contexts of life (e.g., school) needs to be acknowledged and urgently addressed. Such efforts are urgently warranted to reduce the widening of educational and health disparities during the pandemic as well as to build a more equal and inclusive world where minority youth can thrive.

## Data availability statement

The raw data supporting the conclusions of this article will be made available by the authors, without undue reservation.

## Ethics statement

The study protocol and procedures were approved by the ethics committee of the University of Bergen (Norway) (Reference number: 612969). Written informed consent to participate in this study was provided by the participants’ legal guardian/next of kin.

## Author contributions

DM contributed to conception and design of the study, data analysis, interpretation of study findings, and writing the manuscript. SO contributed to manuscript preparation and writing of the manuscript. MK contributed to data analysis and drafting of methods and results. ED, NW, and CR contributed to the interpretation of study findings, and provided feedback of multiple drafts of the manuscript. All authors agreed to byline order and submission of the manuscript in this form, and act as guarantor of the work.

## Funding

The present research project was funded by a grant awarded to DM by the Quebec Population Health Research Network.

## Conflict of interest

The authors declare that the research was conducted in the absence of any commercial or financial relationships that could be construed as a potential conflict of interest.

## Publisher’s note

All claims expressed in this article are solely those of the authors and do not necessarily represent those of their affiliated organizations, or those of the publisher, the editors and the reviewers. Any product that may be evaluated in this article, or claim that may be made by its manufacturer, is not guaranteed or endorsed by the publisher.
