# Supplementary material for: Risk behaviors and well-being among Egyptian and Roma adolescents in Albania during the COVID-19 pandemic: Vulnerability and resilience in a positive youth development perspective
Source: Front Psychol. 2023 Aug 1;14:989661. doi: 10.3389/fpsyg.2023.989661 (PMC10433179; doi:10.3389/fpsyg.2023.989661)
Supplement: Supplementary file 1 [file Table_1.DOCX]

**SUPPLEMENTAL MATERIAL**

Table 1.

*Linear Regression Model Reporting Results of the Interaction of Ethnicity with each of the Main Developmental Assets on Well-being.*

| Variables | Well-being | | | | |
| --- | --- | --- | --- | --- | --- |
|  | Model 1 | Model 2 | Model 3 | Model 4 | Model 5 |
|  | *Coefficient [95% CI]* | *Coefficient [95% CI]* | *Coefficient [95% CI]* | *Coefficient [95% CI]* | *Coefficient [95% CI]* |
| **Age** | -0.04 [-0.10, 0.01] | -0.04 [-0.10, 0.01] | -0.04 [-0.10, 0.01] | -0.04 [-0.10, 0.01] | -0.04 [-0.09, 0.02] |
| **Gender** |  |  |  |  |  |
| Boys |  |  |  |  |  |
| Girls | -0.01 [-0.22, 0.20] | -0.01 [-0.22, 0.21] | -0.02 [-0.23, 0.20] | -0.01 [-0.23, 0.20] | -0.06 [-0.27, 0.15] |
| **Education group** |  |  |  |  |  |
| No education/drop out |  |  |  |  |  |
| At school/completed school | -0.07 [-0.34, 0.21] | -0.04 [-0.32, 0.23] | -0.06 [-0.33, 0.21] | -0.05 [-0.32, 0.23] | -0.07 [-0.34, 0.20] |
| **Parental education** |  |  |  |  |  |
| No education/drop out |  |  |  |  |  |
| At least one parent completed primary education | 0.08 [-0.24, 0.40] | 0.06 [-0.26, 0.38] | 0.07 [-0.25, 0.39] | 0.06 [-0.26, 0.38] | 0.08 [-0.23, 0.40] |
| Both parents completed primary education | 0.25 [-0.06, 0.55] | 0.24 [-0.06, 0.55] | 0.26 [-0.05, 0.57] | 0.27 [-0.04, 0.57] | 0.16 [-0.14, 0.47] |
| **Ethnicity** |  |  |  |  |  |
| Roma |  |  |  |  |  |
| Egyptian | -0.34 [-1.45, 0.77] | 0.19 [-0.65, 1.04] | 0.26 [-0.70, 1.22] | 0.51 [-0.58, 1.61] | -1.08^*^ [-1.97, -0.18] |
| **Family assets** | -0.01 [-0.32, 0.29] | 0.05 [-0.21, 0.30] | 0.05 [-0.20, 0.31] | 0.05 [-0.20, 0.31] | 0.08 [-0.17, 0.33] |
| **Neighbourhood assets** | -0.05 [-0.31, 0.20] | -0.01 [-0.33, 0.32] | -0.06 [-0.31, 0.20] | -0.07 [-0.33, 0.18] | -0.03 [-0.28, 0.22] |
| **Social competencies** | -0.05 [-0.37, 0.27] | -0.04 [-0.36, 0.28] | 0.004 [-0.37, 0.38] | -0.06 [-0.38, 0.26] | -0.12 [-0.43, 0.20] |
| **Positive values** | -0.01 [-0.36, 0.33] | -0.02 [-0.37, 0.33] | -0.02 [-0.36, 0.33] | 0.11 [-0.32, 0.54] | 0.12 [-0.23, 0.48] |
| **Positive identity** | 0.40^**^ [0.14, 0.66] | 0.38^**^ [0.12, 0.65] | 0.40^**^ [0.13, 0.66] | 0.38^**^ [0.11, 0.64] | 0.08 [-0.27, 0.43] |
| **Ethnicity X Family assets** | 0.16 [-0.31, 0.62] |  |  |  |  |
| **Ethnicity X Neighbourhood total** |  | -0.11 [-0.61, 0.40] |  |  |  |
| **Ethnicity X Social competencies** |  |  | -0.10 [-0.50, 0.30] |  |  |
| **Ethnicity X Positive values** |  |  |  | -0.22 [-0.70, 0.26] |  |
| **Ethnicity X Positive identity** |  |  |  |  | 0.57^*^ [0.12, 1.01] |
| *Note.* | ^*^*p*<0.05; ^**^*p*<0.01; ^***^*p*<0.001 | | | | |

Table 2.

*Linear Regression Model Reporting Results of the Interaction of Gender with each of the Main Developmental Assets on Well-being.*

| Variables | Well-being | | | | |
| --- | --- | --- | --- | --- | --- |
|  | Model 1 | Model 2 | Model 3 | Model 4 | Model 5 |
|  | Coefficient [95% CI] | Coefficient [95% CI] | Coefficient [95% CI] | Coefficient [95% CI] | Coefficient [95% CI] |
| **Age** | -0.04 [-0.10, 0.01] | -0.04 [-0.10, 0.01] | -0.04 [-0.10, 0.01] | -0.04 [-0.10, 0.01] | -0.04 [-0.10, 0.01] |
| **Gender** |  |  |  |  |  |
| Boys |  |  |  |  |  |
| Girls | 0.04 [-1.14, 1.23] | 0.27 [-0.57, 1.12] | -0.21 [-1.20, 0.79] | 0.11 [-0.99, 1.22] | 0.13 [-0.76, 1.01] |
| **Education group** |  |  |  |  |  |
| No education/drop out |  |  |  |  |  |
| At school/completed school | -0.05 [-0.33, 0.22] | -0.04 [-0.32, 0.23] | -0.05 [-0.32, 0.22] | -0.06 [-0.33, 0.22] | -0.05 [-0.33, 0.22] |
| **Parental education** |  |  |  |  |  |
| No education/drop out |  |  |  |  |  |
| At least one parent completed primary education | 0.06 [-0.26, 0.39] | 0.06 [-0.26, 0.38] | 0.07 [-0.25, 0.39] | 0.07 [-0.25, 0.39] | 0.06 [-0.26, 0.38] |
| Both parents completed primary education | 0.24 [-0.06, 0.55] | 0.24 [-0.06, 0.54] | 0.24 [-0.06, 0.54] | 0.24 [-0.06, 0.54] | 0.25 [-0.06, 0.55] |
| **Ethnicity** |  |  |  |  |  |
| Roma |  |  |  |  |  |
| Egyptian | 0.02 [-0.22, 0.26] | 0.02 [-0.22, 0.26] | 0.02 [-0.21, 0.26] | 0.02 [-0.21, 0.26] | 0.02 [-0.21, 0.26] |
| **Family assets** | 0.06 [-0.28, 0.39] | 0.04 [-0.22, 0.29] | 0.05 [-0.21, 0.30] | 0.04 [-0.21, 0.30] | 0.04 [-0.21, 0.30] |
| **Neighbourhood assets** | -0.05 [-0.30, 0.20] | 0.03 [-0.31, 0.38] | -0.06 [-0.31, 0.20] | -0.05 [-0.30, 0.21] | -0.05 [-0.30, 0.20] |
| **Social competencies** | -0.04 [-0.36, 0.28] | -0.04 [-0.36, 0.28] | -0.07 [-0.42, 0.28] | -0.04 [-0.36, 0.28] | -0.05 [-0.38, 0.27] |
| **Positive values** | -0.01 [-0.35, 0.34] | -0.001 [-0.35, 0.34] | -0.02 [-0.36, 0.33] | 0.02 [-0.39, 0.42] | -0.001 [-0.35, 0.35] |
| **Positive identity** | 0.39** [0.13, 0.65] | 0.38** [0.12, 0.65] | 0.39** [0.13, 0.65] | 0.39** [0.13, 0.65] | 0.42* [0.09, 0.74] |
| **Gender: X Family assets** | -0.02 [-0.51, 0.47] |  |  |  |  |
| **Gender X Neighbourhood assets** |  | -0.17 [-0.66, 0.32] |  |  |  |
| **Gender X Social competencies** |  |  | 0.08 [-0.32, 0.49] |  |  |
| **Gender X Positive values** |  |  |  | -0.05 [-0.52, 0.42] |  |
| **Gender X Positive identity** |  |  |  |  | -0.07 [-0.49, 0.35] |
| *Note.* | ^*^*p*<0.05; ^**^*p*<0.01; ^***^*p*<0.001 | | | | |

Table 3.

*Linear Regression Model Reporting Results of the Interaction of Education Group with each of the Main Developmental Assets on Well-being.*

| Variables | Well-being | | | | |
| --- | --- | --- | --- | --- | --- |
|  | Model 1 | Model 2 | Model 3 | Model 4 | Model 5 |
|  | Coefficient [95% CI] | Coefficient [95% CI] | Coefficient [95% CI] | Coefficient [95% CI] | Coefficient [95% CI] |
| **Age** | -0.04 [-0.10, 0.01] | -0.04 [-0.10, 0.01] | -0.04 [-0.10, 0.02] | -0.04 [-0.10, 0.01] | -0.04 [-0.10, 0.02] |
| **Gender** |  |  |  |  |  |
| Boys |  |  |  |  |  |
| Girls | -0.01 [-0.22, 0.20] | -0.02 [-0.23, 0.20] | 0.01 [-0.21, 0.22] | -0.004 [-0.22, 0.21] | -0.01 [-0.22, 0.20] |
| **Education group** |  |  |  |  |  |
| No education/drop out |  |  |  |  |  |
| At school/completed school | 0.04 [-1.13, 1.21] | -0.33 [-1.23, 0.57] | -0.58 [-1.57, 0.40] | -0.34 [-1.45, 0.78] | -0.64 [-1.56, 0.28] |
| **Parental education** |  |  |  |  |  |
| No education/drop out |  |  |  |  |  |
| At least one parent completed primary education | 0.06 [-0.26, 0.38] | 0.08 [-0.24, 0.40] | 0.06 [-0.26, 0.38] | 0.07 [-0.25, 0.40] | 0.08 [-0.24, 0.40] |
| Both parents completed primary education | 0.24 [-0.06, 0.55] | 0.25 [-0.05, 0.55] | 0.23 [-0.07, 0.53] | 0.23 [-0.07, 0.54] | 0.25 [-0.06, 0.55] |
| **Ethnicity** |  |  |  |  |  |
| Roma |  |  |  |  |  |
| Egyptian | 0.02 [-0.21, 0.26] | 0.01 [-0.23, 0.25] | 0.02 [-0.22, 0.26] | 0.02 [-0.22, 0.26] | -0.001 [-0.24, 0.24] |
| **Family total** | 0.06 [-0.24, 0.36] | 0.04 [-0.21, 0.30] | 0.04 [-0.21, 0.30] | 0.04 [-0.21, 0.30] | 0.05 [-0.21, 0.30] |
| **Neighbourhood assets** | -0.05 [-0.30, 0.20] | -0.11 [-0.43, 0.21] | -0.03 [-0.28, 0.22] | -0.04 [-0.29, 0.22] | -0.04 [-0.29, 0.21] |
| **Social competencies** | -0.05 [-0.37, 0.27] | -0.04 [-0.36, 0.28] | -0.17 [-0.55, 0.22] | -0.04 [-0.36, 0.28] | -0.09 [-0.41, 0.24] |
| **Positive values** | -0.01 [-0.35, 0.34] | 0.004 [-0.34, 0.35] | 0.02 [-0.33, 0.37] | -0.06 [-0.47, 0.34] | 0.07 [-0.29, 0.43] |
| **Positive identity** | 0.39** [0.13, 0.65] | 0.38** [0.12, 0.64] | 0.37** [0.11, 0.63] | 0.39** [0.13, 0.65] | 0.22 [-0.14, 0.58] |
| **Education X Family assets** | -0.04 [-0.52, 0.44] |  |  |  |  |
| **Education X Neighbourhood assets** |  | 0.16 [-0.35, 0.68] |  |  |  |
| **Education X Social competencies** |  |  | 0.23 [-0.18, 0.64] |  |  |
| **Education X Positive values** |  |  |  | 0.12 [-0.35, 0.60] |  |
| **Education X Positive identity** |  |  |  |  | 0.29 [-0.15, 0.74] |
| *Note.* | ^*^*p*<0.05; ^**^*p*<0.01; ^***^*p*<0.001 | | | | |

Table 4.

*Poisson Regression Model Reporting Results of the Interaction of Ethnicity with each of the Main Developmental Assets on Total Risk Behaviors.*

| Variables | Total risk behaviors | | | | |
| --- | --- | --- | --- | --- | --- |
|  | Model 1 | Model 2 | Model 3 | Model 4 | Model 5 |
|  | Coefficient [95% CI] | Coefficient [95% CI] | Coefficient [95% CI] | Coefficient [95% CI] | Coefficient [95% CI] |
| **Age** | 0.06** [0.02, 0.11] | 0.07** [0.02, 0.11] | 0.06** [0.02, 0.11] | 0.07** [0.02, 0.11] | 0.07** [0.02, 0.11] |
| **Gender** |  |  |  |  |  |
| Boys |  |  |  |  |  |
| Girls | -1.1** [-1.29, -0.92] | -1.1** [-1.28, -0.91] | -1.12** [-1.3, -0.93] | -1.11** [-1.29, -0.93] | -1.12** [-1.31, -0.93] |
| **Education group** |  |  |  |  |  |
| No education/drop out |  |  |  |  |  |
| At school/completed school | -0.01 [-0.19, 0.17] | -0.02 [-0.19, 0.16] | -0.06 [-0.24, 0.12] | -0.04 [-0.22, 0.14] | -0.04 [-0.22, 0.14] |
| **Parental education** |  |  |  |  |  |
| No education/drop out |  |  |  |  |  |
| At least one parent completed primary education | -0.11 [-0.33, 0.11] | -0.08 [-0.29,0.14] | -0.06 [-0.28,0.15] | -0.08 [-0.29, 0.14] | -0.08 [-0.29, 0.13] |
| Both parents completed primary education | -0.12 [-0.32, 0.08] | -0.12 [-0.33, 0.08] | -0.09 [-0.3,0.12] | -0.09 [-0.3, 0.12] | -0.15 [-0.36, 0.05] |
| **Ethnicity** |  |  |  |  |  |
| Roma |  |  |  |  |  |
| Egyptian | 1.01* [0.36, 1.65] | 0.45 [-0.02, 0.92] | 0.44 [-0.31, 1.18] | 0.52 [-0.37, 1.41] | -0.24 [-0.92, 0.44] |
| **Family assets** | 0.27* [0.05, 0.5] | 0.10 [-0.06, 0.26] | 0.12 [-0.04, 0.29] | 0.12 [-0.05, 0.28] | 0.12 [-0.04, 0.27] |
| **Neighbourhood assets** | -0.02 [-0.17, 0.13] | 0.09 [-0.12, 0.29] | -0.03 [-0.18, 0.12] | -0.04 [-0.2, 0.11] | -0.02 [-0.17, 0.13] |
| **Social competencies** | 0.19 [-0.04, 0.42] | 0.19 [-0.04, 0.42] | 0.27 [-0.01, 0.55] | 0.17 [-0.06, 0.4] | 0.16 [-0.07, 0.39] |
| **Positive values** | -0.08 [-0.32, 0.16] | -0.12 [-0.36, 0.12] | -0.13 [-0.38, 0.13] | 0.02 [-0.3, 0.34] | -0.06 [-0.32, 0.2] |
| **Positive identity** | -0.05 [-0.27, 0.16] | -0.04 [-0.25, 0.18] | -0.02 [-0.23, 0.2] | -0.04 [-0.26, 0.17] | -0.11 [-0.4, 0.19] |
| **Ethnicity X Family assets** | -0.43* [-0.72, -0.14] |  |  |  |  |
| **Ethnicity X Neighbourhood assets** |  | -0.26 [-0.54, 0.03] |  |  |  |
| **Ethnicity X Social competencies** |  |  | -0.17 [-0.48, 0.13] |  |  |
| **Ethnicity X Positive values** |  |  |  | -0.22 [-0.62, 0.17] |  |
| **Ethnicity X Positive identity** |  |  |  |  | 0.14 [-0.2, 0.49] |
| *Note.* | ^*^*p*<0.05; ^**^*p*<0.01; ^***^*p*<0.001 | | | | |

Table 5.

*Poisson Regression Model Reporting Results of the Interaction of Gender with each of the Main Developmental Assets on Total Risk Behaviors.*

| Variables | Total risk behaviors | | | | |
| --- | --- | --- | --- | --- | --- |
|  | Model 1 | Model 2 | Model 3 | Model 4 | Model 5 |
|  | Coefficient [95% CI] | Coefficient [95% CI] | Coefficient [95% CI] | Coefficient [95% CI] | Coefficient [95% CI] |
| **Age** | 0.07** [0.02, 0.11] | 0.06** [0.01, 0.11] | 0.06** [0.02, 0.11] | 0.06** [0.02, 0.11] | 0.07** [0.02, 0.11] |
| **Gender** |  |  |  |  |  |
| Boys |  |  |  |  |  |
| Girls | -1.38** [-2.45, -0.3] | -1.75** [-2.41, -1.09] | -1.9** [-2.84, -0.97] | -2.08** [-3.14, -1.03] | -1.13** [-2.06, -0.2] |
| **Education group** |  |  |  |  |  |
| No education/drop out |  |  |  |  |  |
| At school/completed school | -0.04 [-0.21, 0.14] | -0.06 [-0.24, 0.12] | -0.03 [-0.2, 0.15] | -0.03 [-0.21, 0.15] | -0.04 [-0.22, 0.14] |
| **Parental education** |  |  |  |  |  |
| No education/drop out |  |  |  |  |  |
| At least one parent completed primary education | -0.07 [-0.29, 0.14] | -0.07 [-0.28, 0.14] | -0.06 [-0.28, 0.15] | -0.08 [-0.29, 0.14] | -0.08 [-0.29, 0.14] |
| Both parents completed primary education | -0.12 [-0.33, 0.09] | -0.12 [-0.32, 0.09] | -0.13 [-0.33, 0.08] | -0.11 [-0.32, 0.09] | -0.12 [-0.33, 0.08] |
| **Ethnicity** |  |  |  |  |  |
| Roma |  |  |  |  |  |
| Egyptian | 0.03 [-0.13, 0.2] | 0.04 [-0.12, 0.2] | 0.04 [-0.12, 0.2] | 0.03 [-0.13, 0.19] | 0.03 [-0.13, 0.2] |
| **Family assets** | 0.08 [-0.07, 0.24] | 0.13 [-0.04, 0.29] | 0.12 [-0.05, 0.28] | 0.12 [-0.04, 0.28] | 0.11 [-0.05, 0.27] |
| **Neighbourhood assets** | -0.02 [-0.17, 0.13] | -0.12 [-0.29, 0.05] | -0.05 [-0.2, 0.1] | -0.05 [-0.2, 0.1] | -0.02 [-0.17, 0.13] |
| **Social competencies** | 0.18 [-0.05, 0.41] | 0.17 [-0.05, 0.40] | 0.13 [-0.1, 0.35] | 0.17 [-0.06, 0.41] | 0.18 [-0.05, 0.41] |
| **Positive values** | -0.1 [-0.33, 0.14] | -0.11 [-0.36, 0.13] | -0.12 [-0.37, 0.12] | -0.19 [-0.44, 0.06] | -0.1 [-0.34, 0.14] |
| **Positive identity** | -0.03 [-0.24, 0.19] | -0.03 [-0.24, 0.19] | -0.02 [-0.23, 0.19] | -0.03 [-0.24, 0.19] | -0.03 [-0.25, 0.19] |
| **Gender X Family assets** | 0.11 [-0.32, 0.54] |  |  |  |  |
| **Gender X Neighbourhood assets** |  | 0.38 [0.01, 0.76] |  |  |  |
| **Gender X Social competencies** |  |  | 0.32 [-0.04, 0.68] |  |  |
| **Gender X Positive values** |  |  |  | 0.41 [-0.01, 0.83] |  |
| **Gender X Positive identity** |  |  |  |  | 0.01 [-0.43, 0.45] |
| *Note.* | ^*^*p*<0.05; ^**^*p*<0.01; ^***^*p*<0.001 | | | | |

Table 6.

*Poisson Regression Model Reporting Results of the Interaction of Education Group with each of the Main Developmental Assets on Total Risk Behaviors.*

| Variables | Total risk behaviors | | | | |
| --- | --- | --- | --- | --- | --- |
|  | Model 1 | Model 2 | Model 3 | Model 4 | Model 5 |
|  | Coefficient [95% CI] | Coefficient [95% CI] | Coefficient [95% CI] | Coefficient [95% CI] | Coefficient [95% CI] |
| **Age** | 0.07** [0.02, 0.11] | 0.06** [0.02, 0.11] | 0.06** [0.02, 0.11] | 0.06** [0.02, 0.11] | 0.07** [0.02, 0.11] |
| **Gender** |  |  |  |  |  |
| Boys |  |  |  |  |  |
| Girls | -1.11** [-1.29, -0.93] | -1.09** [-1.28, -0.91] | -1.13** [-1.31, -0.95] | -1.12** [-1.31, -0.94] | -1.11** [-1.29, -0.93] |
| **Education group** |  |  |  |  |  |
| No education/drop out |  |  |  |  |  |
| At school/completed school | 0.09 [-0.55, 0.73] | 0.41 [-0.09, 0.91] | 0.5 [-0.23, 1.23] | 0.58 [-0.29, 1.45] | -0.26 [-0.97, 0.45] |
| **Parental education** |  |  |  |  |  |
| No education/drop out |  |  |  |  |  |
| At least one parent completed primary education | -0.08 [-0.3, 0.14] | -0.09 [-0.31, 0.12] | -0.07 [-0.29, 0.14] | -0.1 [-0.31, 0.12] | -0.07 [-0.29, 0.15] |
| Both parents completed primary education | -0.12 [-0.33, 0.08] | -0.12 [-0.33, 0.08] | -0.11 [-0.32, 0.09] | -0.11 [-0.32, 0.10] | -0.12 [-0.33, 0.08] |
| **Ethnicity** |  |  |  |  |  |
| Roma |  |  |  |  |  |
| Egyptian | 0.04 [-0.12, 0.2] | 0.05 [-0.11, 0.21] | 0.03 [-0.13, 0.19] | 0.03 [-0.13, 0.19] | 0.03 [-0.14, 0.19] |
| **Family assets** | 0.13 [-0.08, 0.34] | 0.1 [-0.06, 0.26] | 0.11[-0.04, 0.27] | 0.11 [-0.05, 0.27] | 0.11 [-0.05, 0.27] |
| **Neighbourhood assets** | -0.02[-0.17,0.13] | 0.1 [-0.12, 0.31] | -0.04[-0.19, 0.11] | -0.04 [-0.2, 0.11] | -0.02 [-0.17,0.13] |
| **Social competencies** | 0.18[-0.05,0.41] | 0.17 [-0.06, 0.4] | 0.32[0.01, 0.63] | 0.17 [-0.06, 0.40] | 0.17 [-0.06, 0.4] |
| **Positive values** | -0.1[-0.34,0.14] | -0.12 [-0.36, 0.12] | -0.13[-0.38, 0.12] | 0.05 [-0.27, 0.36] | -0.07 [-0.33, 0.19] |
| **Positive identity** | -0.03[-0.24,0.19] | -0.02 [-0.23, 0.19] | -0.02[-0.24, 0.19] | -0.05 [-0.26, 0.17] | -0.1 [-0.43, 0.23] |
| **School X Family assets** | -0.05[-0.33,0.22] |  |  |  |  |
| **School X Neighbourhood assets** |  | -0.27 [-0.56, 0.02] |  |  |  |
| **School X Social competencies** |  |  | -0.23 [-0.54, 0.07] |  |  |
| **School X Positive values** |  |  |  | -0.28 [-0.65, 0.10] |  |
| **School: X Positive identity** |  |  |  |  | 0.11 [-0.24, 0.47] |
| *Note.* | ^*^*p*<0.05; ^**^*p*<0.01; ^***^*p*<0.001 | | | | |
